# Towards an understanding of the avian virome

**DOI:** 10.1099/jgv.0.001447

**Published:** 2020-06-10

**Authors:** Sarah François, Oliver G Pybus

**Affiliations:** ^1^​ Department of Zoology, University of Oxford, UK; ^2^​ Department of Pathobiology and Population Sciences, Royal Veterinary College London, UK

**Keywords:** ecology, epidemiology, metagenomics, poultry, virus, wild birds

## Abstract

The last two decades have seen the rise of viromics, the study of viral communities through the detection and characterization of virus genome sequences. Here we systematically review and summarize the scope and limitations of our current understanding of avian viromes, in both domesticated and wild-bird populations. We compare this viromic work to the broader literature on avian prokaryotic microbiomes, and highlight the growing importance of structured sampling and experimental design for testing explanatory hypotheses. We provide a number of recommendations for sample collection and preliminary data analysis to guide the development of avian viromics. Avian viromes have the potential to inform disease surveillance in poultry and improve our understanding of the risk of zoonotic viruses to human health.

## Introduction

The development and adoption of high-throughput sequencing technologies has led to studies of microbial and viral communities (microbiomes and viromes) being undertaken across an increasing range of host species [1]. Among these, studies of bird populations are common, in part due to their potential relevance to the poultry industry. Intensification of the poultry industry over the last century means that the biomass of poultry represents about 70 % of the total biomass of birds worldwide [2]. Poultry flock populations are often high-density and genetically homogenous, potentially rendering them susceptible to outbreaks of viral infectious disease, and dysbiosis of their viromes and microbiomes may impact poultry health and growth. These situations can result in substantial economic losses to the poultry industry and contribute to food insecurity. The viromes of wild birds are also relevant to poultry, as many important pathogens of domesticated birds originate from wild-bird populations, most notably avian influenza A viruses. Further, the majority of zoonotic pathogens in humans are thought to originate from wildlife, and viruses are estimated to represent 75 % of new human pathogens discovered since 1980 [3, 4]. Here we survey the scope and limitations of our current understanding of avian viromes, and we provide recommendations for the future development of this important topic.

### Current understanding of the avian virome

**Fig. 1.**
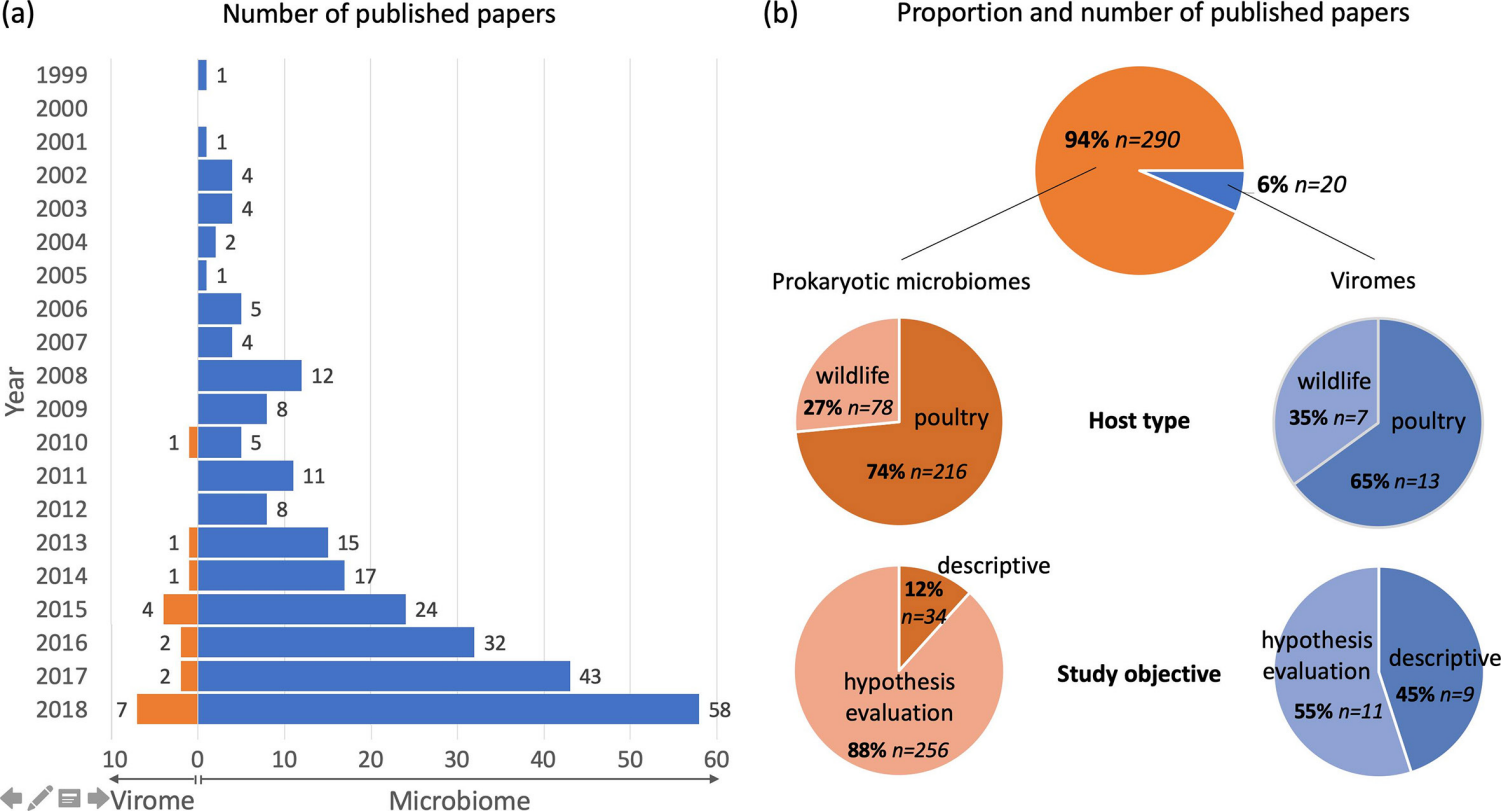
Comparison of research effort between bird viromes and prokaryotic microbiomes. We conducted a bibliographic search of PubMed on 16 September 2019 using the Adjutant package in R. We retrieved papers on bird prokaryotic microbiomes using the search ‘(bird OR avian) AND (bact metagenomic OR 16S OR bact microbio)’ (1424 papers). We retrieved papers on bird viromes using the search ‘(bird OR avian) AND (virome OR vir metagenomic OR vir communit OR vir diversity OR vir composition)’ and supplemented this search by manual screening, resulting in 28 papers. We removed papers (i) that were non-research articles, (ii) whose samples were not taken from living birds, or (iii) that targeted one or few specific virus/prokaryote taxa. We consequently retained a total of 310 papers on bird microbiomes and viromes (full details in Tables S1 and S2). (a) The bar chart and values show the number of papers published each year on bird microbiomes (orange) and bird viromes (blue). (b) The top pie chart shows the total number of published virome and microbiome papers. The other pie charts show the number of papers structured by host type (middle) and by study objective (bottom, see main text for definition of ‘descriptive’ and ‘hypothesis evaluation’). Due to the small number of bird virome studies, the corresponding proportions should be interpreted with caution.

To our knowledge, only 20 research papers concerning the viromes of birds have been published to date. This contrasts with an older, larger and faster-growing body of research on the prokaryotic microbiome of birds, which comprises 290 published research papers published over two decades (Fig. 1, Tables S1 and S2, available in the online version of this article). The large discrepancy in research effort directed towards microbiomes and viromes is not limited to birds [5] and can be explained by both conceptual and technical factors.

Firstly, until quite recently, viruses have been conceptualized primarily as pathogens and not as normal or natural constituents of the host microbiota. The prevailing ‘viruses-as-pathogens’ viewpoint arose from the fact that viruses are obligatory intracellular parasites, combined with a ‘one disease, one microbe’ model of pathogenesis that ignored other potential explanatory factors [6]. As a consequence, avian virus research efforts to date have focussed on investigating a comparatively small number of virus species of economic and animal health importance (especially avian influenza viruses, but also duck plague virus, West Nile virus, infectious bursal disease virus and Newcastle disease virus).

**Fig. 2.**
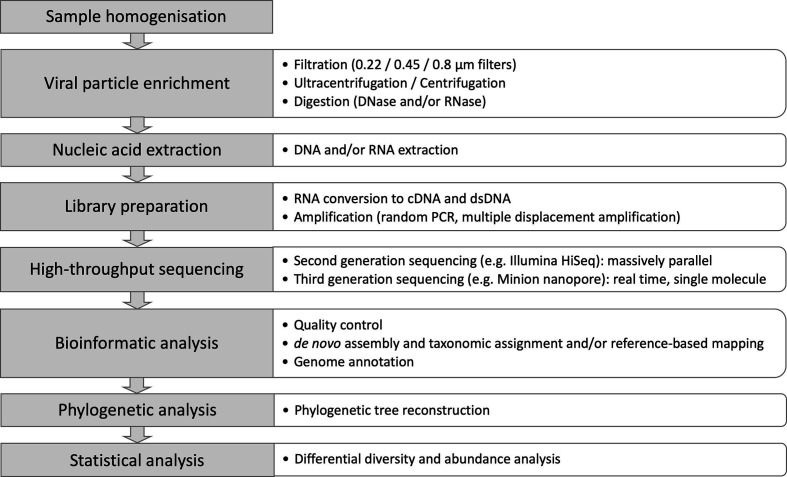
General workflow of VANA virome generation and analysis. The main difference between VANA metagenomics and metatranscriptomics is that the latter is based solely on RNA extraction, without DNA extraction or prior enrichment for viral particles.

Secondly, viruses lack a universal gene marker that can be used for their identification, in contrast to the conserved regions of the 16S rRNA gene that are used routinely to classify prokaryotes at the genus or species level [1]. Further, the low abundance of virus nucleic acids in biological samples, due to the dominance of cellular-derived material, can lead to a low proportion of virus-specific reads in metagenomic analyses of bird samples (between 0.0001 and 1 % [7]. As a consequence, many virome studies choose to undertake one or more virus-specific enrichment steps (e.g. filtration, centrifugation, digestion of non-encapsidated nucleic acids) before proceeding to non-selective DNA amplification. The two main approaches that are used to characterize viromes are (i) metagenomics of nucleic acids associated with virions (VANA metagenomics) and (ii) metatranscriptomics. These are summarized in Fig. 2. An additional technical consideration is the fact that 56 % of viral families (74/131) have an RNA genome [8], necessitating alternative methods of sample preservation and storage and further steps to reliably recover RNA viromes (i.e. conversion to cDNA before sequencing).

Studies of bird viromes are not only much rarer than those of bird microbiomes, they also differ in nature and scope. Bird virome studies are more likely than microbiome studies to be purely descriptive (45 vs 14 % of studies, Fig. 1b), i.e. they catalogue the viruses present in samples, without evaluating hypotheses that might explain the dynamics and diversity of viral communities. Both virome and microbiome studies have been conducted more often in poultry populations than in wild birds (65 vs 74 % of studies; Fig. 1b, Table S1). Five of the seven studies of wild-bird viromes involved waterbirds (e.g. ducks, teals, sandpipers), one of which involved an endangered species (the red-crowned crane). Most studies investigated viromes of the gastro-intestinal tract or faeces (90 % of studies), or of the respiratory tract (15 % of studies). Viromes of skin, mesenteric fat, pancreas or proventriculi were described only once each.

The viromes of healthy, asymptomatic birds appear to contain many diverse bird-infecting viruses. Virus genetic sequences belonging to the families *Parvoviridae*, *Picornaviridae*, *Circoviridae*, *Reoviridae*, *Caliciviridae*, *Adenoviridae*, *Picobirnaviridae* and *Astroviridae* seem to be frequent inhabitants of birds’ intestinal tracts and faeces, and reported by the majority of virome studies with available abundance data. Members of the families *Herpesviridae*, *Orthomyxoviridae*, *Coronaviridae*, *Poxviridae*, *Anelloviridae* and *Paramyxoviridae* were reported by at least one-third of studies, whilst sequences belonging to the families *Flaviviridae*, *Rhabdoviridae*, *Hepadnaviridae*, *Polyomaviridae*, *Papillomaviridae*, *Birnaviridae*, *Hepeviridae*, *Arteriviridae* and *Bornaviridae* were reported in one or two studies. In terms of the abundance of bird-infecting viruses, gastro-intestinal and faecal viromes are dominated by members of the *Picornaviridae* and *Parvoviridae* families (accounting for ≈30 % of virus reads), whilst members of families *Orthomyxoviridae*, *Herpesviridae*, *Reoviridae*, *Astroviridae*, *Circoviridae*, *Adenoviridae*, *Coronaviridae*, *Caliciviridae*, *Retroviridae* and *Poxviridae* together account for ≈50 % of virus reads. Almost all avian virome studies reported multiple new virus species, mostly from the *Picornaviridae* (*n*=17), *Parvoviridae* (7), *Circoviridae* (6), *Caliciviridae* (5) and *Reoviridae* (4) families. Some of these viruses could belong to new genera and potentially could infect birds. These results indicate that we do not yet have a clear idea of the range and diversity of viruses that infect poultry, and our knowledge of viruses infecting wild birds is even scanter.

It is important to remember that virus genetic sequences detected in virome studies can be derived from a range of sources. For example, five of nine studies that reported retroviral sequences in bird samples hypothesized that those sequences originated from endogenous retroviruses; two such studies concluded showed that endogenized viruses can represent a high proportion of *Retroviridae* sequences [9, 10]. Endogenous viral sequences derived from a wide range of virus families are present in avian genomes [11] and they should be kept in mind whilst interpreting virome data, as they can have a significant impact on viral community structure and composition. Avian viromes also include virus sequences from bacteriophages, the environment, and diet-associated viruses, all of which are to be expected in the output of untargeted metagenomic sequencing. Further, modern poultry farming uses live vaccines for disease control and live-attenuated viruses that might be found in poultry viromes. Only 2 of 20 studies mentioned this hypothesis, both of which reported the presence of virus sequences from live-attenuated vaccines in poultry [9, 12]. Such sequences are detectable in poultry viromes at least 18 weeks post-vaccination [9], however, it is unclear whether the viromic detection of such sequences is linked or not to the shedding of infectious virus, or to transmission to wildlife. One study detected viruses sequences that were genetically similar to Newcastle disease virus vaccine strains in 17 different species of wild birds across four continents and over >15 years [13]. The reverse spillover of viruses from domestic animals to wildlife is expected to increase in the future, yet they represent an under-appreciated and poorly studied consequence of human activity on wild birds.

Beyond these observations, no further trends or conclusions can be drawn, in particular concerning the comparison of poultry and wild-bird viromes, due to the small number avian virome studies that have been published (even for poultry populations), and the small number of bird species investigated (26 to date). Numerous differences in metagenomic protocols and study designs also preclude detailed meta-analysis.

### Prospects for future research

Clearly, further virus discovery studies are required to improve our knowledge of the diversity of viruses in birds. As well as expanding the inventory of known bird viruses, the field of avian viromics would benefit from a greater focus on fundamental questions that recognize the ubiquity and varied roles of viruses in hosts and their ecosystems. Some important research questions can be identified from the existing literature on microbial ecology [14], for example, the following.

- Which viruses are normal constituents of healthy bird viromes, and which are not? What factors cause viruses to switch from asymptomatic carriage to pathogenic infection?
- What mechanisms drive the structure and functions of virus communities in birds? Are stochastic or deterministic processes more important? What are the relative contributions of host-specific versus environment-specific factors in shaping bird viromes?
- How do bird viromes vary among individuals and populations, through time, and across space?

Several of these topics have been already investigated in mammal populations [15, 16] and addressing them in birds will benefit both virus ecology and ornithology. However, tackling these questions adequately will require changes to the way in which bird viromes are sampled, sequenced and analysed. In the remainder of this article we outline a series of recommendations for sample collection and virome analysis, which we hope will increase the accuracy, scope and relevance of future bird virome studies.

### Recommendations for sample collection

In order to test biological hypotheses regarding virome composition, samples must be collected in a more structured manner than for virus discovery studies. For example, individual-level information, (instead of pooling samples from different animals) is needed to make inferences about differential prevalence or abundance of viruses among birds in a population. Longitudinal sampling (of the same individuals, if possible) on a timescale determined by the lifespan and life history of the bird species in question will help to elucidate temporal virome changes and to distinguish transient from persistent viruses [17]. Further, sampling across different spatial scales and locations will allow us to test the impact of habitat and geographical distance on the sharing and structure of bird virus communities [18].

It is also important to obtain as much relevant sample metadata as possible. This can be challenging for natural habitats and communities that contain a broad range of bird species at different population densities, particularly if sampling is non-invasive (e.g. from faeces). Wild birds may vary in age, sex, genotype and immune status, so avian viromics will benefit from collaborations with ornithologists who have expertise in handling and measurement of wild birds and expert knowledge of their ecology and demography.

Environmental factors can influence the persistence of and transmission of viruses outside their hosts. For example, salinity and temperature affect the persistence of avian influenza viruses in water [19]. It is therefore valuable to measure environmental variables and collect samples from bird habitats when appropriate, especially samples of the water column in the case of waterbirds.

Lastly, sampling effort must be sufficient to allow statistical analysis and testing of the hypotheses under investigation [20, 21], particularly when the objective is to obtain information on plausible causative agents of diseases of unknown aetiology, such as bird enteritis diseases [22], runting-stunting syndrome [10] or malabsorption syndrome [23].

### Recommendations for virome analysis

Viromes in natural populations will be characterized best by minimising possible biases in virus composition arising from sample storage, enrichment, sequencing and processing. The efficiencies of many protocols and pipelines have been compared since the publication of early VANA metagenomic approaches (e.g. [24]). As a result, standardized methods are now available for obtaining and analysing viromic data, from sample transport and preparation, to bioinformatic and statistical analysis (see [25, 26]).

Viral genetic material is present in low concentration in biological samples, even after ultracentrifugation and digestion steps, because of the overwhelming abundance of cellular nucleic acids [27]. Increasing the relative abundance of virus sequences is therefore needed (i) to recover sufficient virus sequence diversity to allow analysis of the virus community, and (ii) to reconstruct full-length virus genomes. The optimal sequencing depth can be assessed by conducting a preliminary sequencing round, followed by the construction of rarefaction curves [28].

The incorporation of negative controls is an important step, as high-throughput sequencing is sensitive to contamination by virus genetic sequences present not only on skin but also in laboratory reagents that are thought to be sterile [29, 30]. Cross-sample contamination during sample processing and library preparation [31, 32] is a further risk that can be reduced by careful experimental design and bioinformatic analysis [33]. One or more positive controls (representing sequences that are not expected in bird viromes) can be ‘spiked-in’ at known concentrations to help quantify the level of cross-contamination as well as the relationship between viral read abundance and virus concentration in samples [34].

Viruses belong to host microbial communities, and some viruses have been shown to influence the diversity and composition of birds’ bacterial communities. This interaction is not limited to bacteriophages, as illustrated by seven papers that highlight the impact of bird viruses (e.g. avian influenza, Avian leukosis, Haemorrhagic enteritis and Marek's disease viruses) on prokaryotic microbial diversity and composition (Table S2). Thus, joint analyses of viromes and prokaryotic microbiomes in the same host population will be of great interest. To our knowledge, only one study has carried out this direct comparison, in broiler chickens [35].

Finally we suggest that, in addition to submitting reconstructed viral contigs to GenBank, researchers also place the raw sequencing data into open repositories such as Sequence Read Archive (SRA) [36], together along with metadata on experimental design, sequencing methods and data processing (as listed by [37]. This will facilitate meta-analyses and the discovery of new virus groups.

### Conclusion

Our understanding of avian viromes is in its infancy. However, decreasing sequencing costs, coupled with increasing sequencing depth and standardized protocols and pipelines, offer the opportunity to obtain and analyse unprecedented amounts of data [26, 38]. Through collaborations among virologists, bacteriologists and ecologists, an improved characterization of bird viromes will increase our understanding of virus ecology and evolution. This knowledge could be used to improve poultry health and to aid conservation, by mitigating viral outbreaks in endangered birds (which represent about 14 % of the all bird species [39]). Ultimately, bird viromes could be used to inform and design appropriate surveillance programmes to prevent and mitigate the effects to human of future zoonoses, which are likely to continue to occur due to increasing contact between humans and wildlife [40].

## Supplementary Data

Supplementary material 1
